# MRI appearance of Bochdalek's flower basket in a patient with bilateral hippocampus infarct

**DOI:** 10.1016/j.radcr.2024.11.024

**Published:** 2024-12-07

**Authors:** Anshul Sood, Gaurav Vedprakash Mishra, Shreya Khandelwal, Nishtha Manuja, Suhit Naseri

**Affiliations:** a Department of Radiodiagnosis, Jawaharlal Nehru Medical College, Datta Meghe Institute of Higher Education and Research, Sawangi (Meghe), Wardha, Maharashtra, India, 442001; b Department of Medicine, Jawaharlal Nehru Medical College, Datta Meghe Institute of Higher Education and Research, Sawangi (Meghe), Wardha, Maharashtra, India, 442001; c Department of Pathology, Jawaharlal Nehru Medical College, Datta Meghe Institute of Higher Education and Research, Sawangi (Meghe), Wardha, Maharashtra, India, 442001

**Keywords:** Hippocampus infarct, Bochdalek flower basket, Choroid plexus variant, MRI, Radiology, Acute infarct

## Abstract

The occurrence of isolated bilateral hippocampus infarct is extremely rare and is thought to be associated with a number of etiologies, including ischemia, infection, paraneoplastic syndromes, seizures, drug addiction, etc. The presented manuscript depicts a case of a 28-year-old male patient who has been a chronic alcoholic for the past 4 years and, on imaging, was found to have a bilateral hippocampal infarct in isolation. Also, the manuscript presents the appearance of the magnetic resonance imaging (MRI) of Bochdalek's flower basket, which is an anatomical variant of the choroid plexus in the fourth ventricle.

## Case report

A 28-year-old male patient with a daily drinking habit for the past 4 years, with the last intake one day ago, was brought to the emergency department by his relative, who noticed that the patient did not get up in the morning. On examination, the patient was unconscious with neck rigidity and a GCS of E1VTM3. He did not have any history of drug addiction, including cocaine and opioids, no history of seizures or loss of consciousness, and no known systemic illness.

**Fig. 1:**
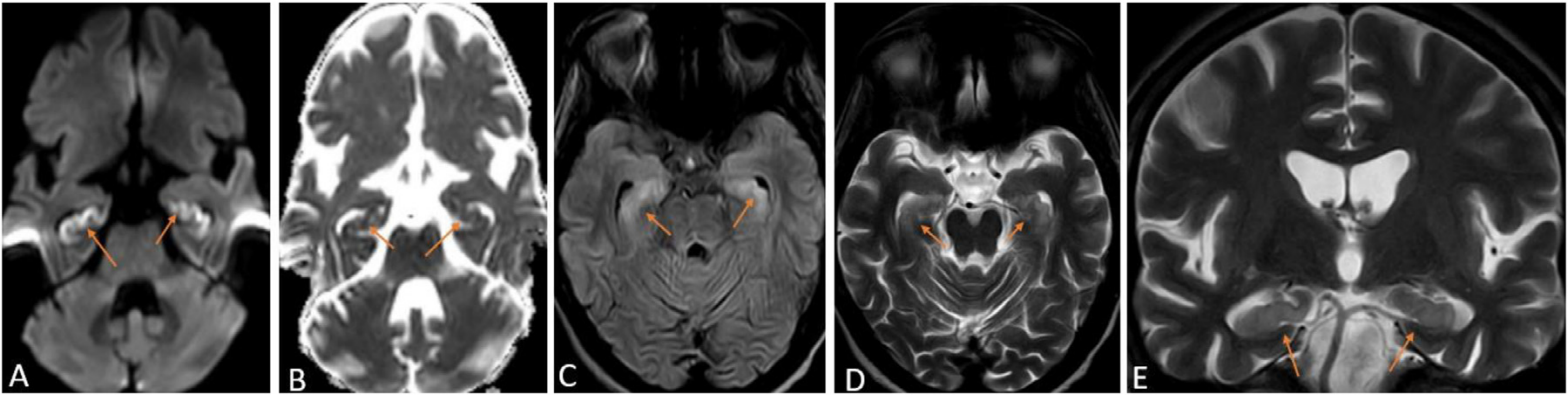
Magnetic Resonance Imaging of the brain of a 28-year-old chronic alcoholic with a history of loss of consciousness after sleeping in the night axial section Diffusion-weighted imaging (A), Apparent diffusion coefficient (B) Fluid attenuation inversion recovery sequence (C), T2 weighted imaging (D), and coronal section T2 weighted imaging (E) showing bilateral hippocampal areas showing restricted diffusion with corresponding low signals on ADC and increased T2/FLAIR signal intensity consistent with edema suggesting bilateral hippocampal infarcts (orange arrows).

**Fig. 2:**
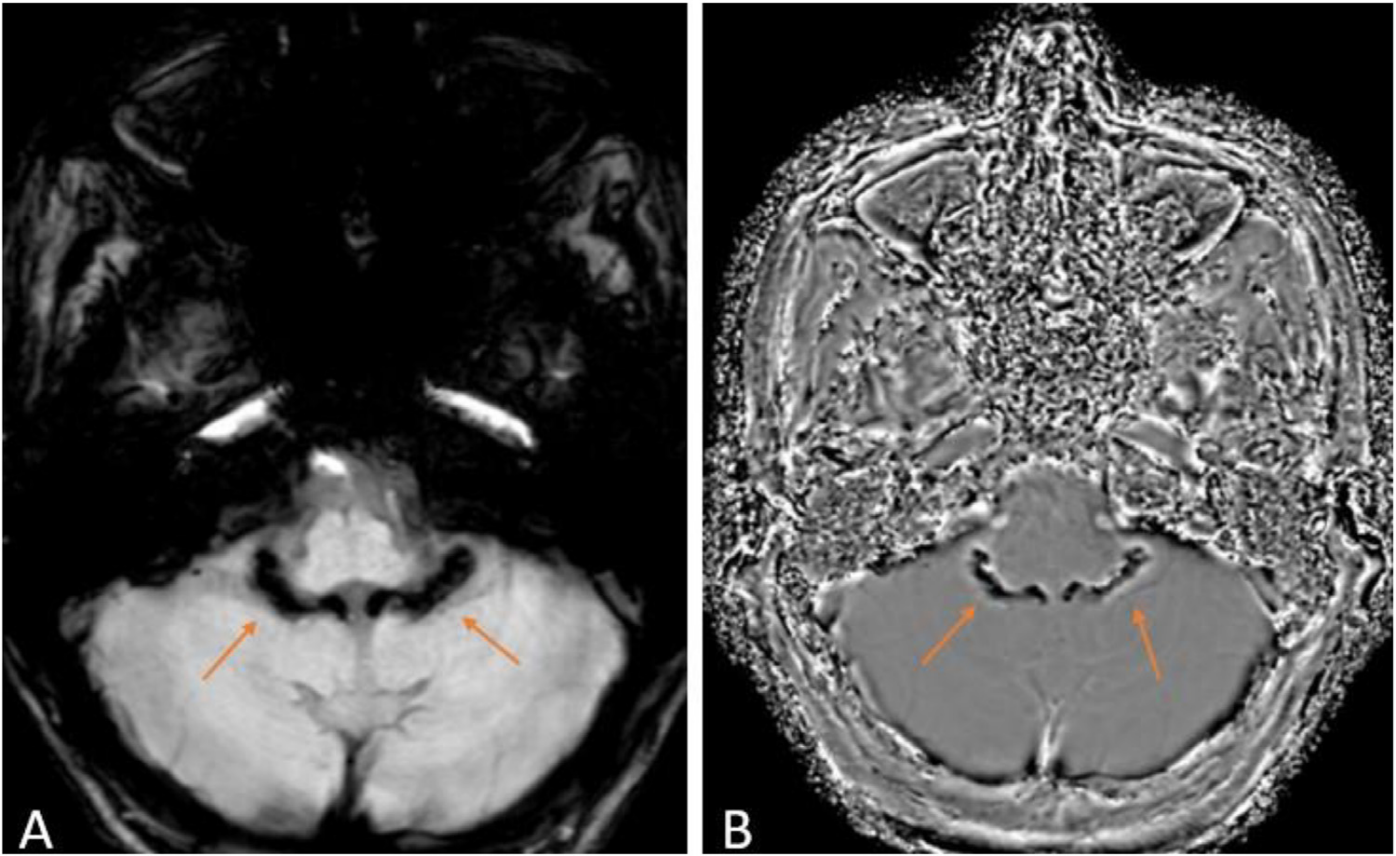
Magnetic resonance imaging of the brain of a 28-year-old chronic alcoholic with a history of loss of consciousness after sleeping in the night, axial section susceptibility-weighted imaging phase (A) and susceptibility-weighted imaging magnitude (B), showing Bochdalek's flower basket – a normal anatomical variant of choroid plexus (orange arrows).

The patient's blood tests and cerebrospinal fluid analysis were done, which did not reveal any significant findings. Magnetic resonance imaging (MRI) of the brain was advised, which revealed isolated bilateral hippocampal acute infarct (Fig. 1) and Bochdalek's flower basket (Fig. 2). The patient was shifted to the intensive care unit (ICU), and anti-platelet therapy was started. The patient was extubated on the sixth day of admission. He was confused and had difficulty immediately recognizing and naming anything or anyone and was being helped by his relatives. The speech was not clear, and he had to put effort into speaking. A psychiatric call was made, which revealed that the patient had partial anterograde amnesia, which was causing him depression. However, there were no effects on long-term memories before the hospitalization. His relatives were advised to support him through the amnesia. The vitals were regularly monitored, and once stable, the patient was discharged with advice to follow up after 15 days or if any complaints developed. The GCS was E4V5M6 at the time of discharge, with slightly slurry speech, and the power in all his limbs was 4/5.

## Discussion

The hippocampus is an important part of the limbic system, which plays a crucial role in the formation of memory and helps in recall [1]. It is situated in the watershed area, where the arterial supply is made by the anterior, posterior, and middle hippocampal arteries. Isolated bilateral hippocampal involvement is a rare pathology with a reported incidence of 0.03% [2] and is thought to be caused by varied etiologies involving ischemia, infection, seizures, paraneoplastic syndromes, and drugs like cocaine and opioids [3,4]. It is of paramount importance to obtain a proper medical history to rule out the cause of the infarction. The patient might present with complaints of cognitive deterioration and sudden memory loss resembling a transient global amnesia-like episode [5]. MRI remains the imaging modality of choice. The prognosis for bilateral lesions is worse than that of unilateral lesions and is reported to be associated with more severe amnesia [6]. The differential diagnosis includes limbic encephalitis, which shows hippocampal edema without restricted diffusion [2].

Small et al. [2] conducted a study in a tertiary care center over a period of 3 years, which involved 4 patients with acute ischemia of hippocampi, and concluded the presence of a common link between hippocampal ischemia and toxic substance abuse. In a study done by Bhattacharyya et al. [7] on 16 patients with bilateral diffusion restriction in the area of the hippocampus with or without the involvement of the thalamus, basal ganglia, or cerebellum, it was concluded that all the surviving patients remained amnestic for up to 20 months.

Bochdalek's flower basket is a rare appearance for people in their 20s and is mostly found in the adult population. It is characterized by the presence of linear choroid plexus calcification in the fourth ventricle protruding through paired Luschka foramina. They might show a linear or bulbous appearance and could be misinterpreted as a subarachnoid hemorrhage or a mass lesion, respectively. Hence, it is important to have an adequate knowledge of the choroid plexus variant to prevent misdiagnosis [8].

## Patient consent

An informed verbal and written consent was obtained from the patient.
